# Cross-cultural adaptation and semantic validation of an instrument to identify palliative requirements in Portuguese

**DOI:** 10.31744/einstein_journal/2020AO5539

**Published:** 2020-10-02

**Authors:** Marcella Tardeli Esteves Angioleti Santana, Xavier Gómez-Batiste, Lucia Marta Giunta da Silva, Maria Gaby Rivero de Gutiérrez

**Affiliations:** 1 Universidade Federal de São Paulo São PauloSP Brazil Universidade Federal de São Paulo, São Paulo, SP, Brazil.; 2 Institut Català d’Oncologia Barcelona Spain Institut Català d’Oncologia, Barcelona, Spain.

**Keywords:** Palliative care, Chronic disease, Patient identification systems, Patient care management, Surveys and questionnaires, Validation study

## Abstract

**Objective:**

To translate and make cross-cultural adaptation of NECPAL CCOMS-ICO^©^ tool to Portuguese, and to analyze its semantic validity.

**Methods:**

A methodological research about NECPAL CCOMS-ICO^©^ tool cross-cultural adaptation, translated from Spanish into Portuguese and measurement of semantic validity. The cross-cultural adaptation process was conducted according to Beaton recommendations, including translation, translation synthesis, back-translation, and analysis of semantic, idiomatic, conceptual, and cultural equivalence of translated and back-translated tool versions, resulting in a pre-final version, which was submitted to a pre-test (n=35). Contend Validity Index was calculated to analyze semantic validity.

**Results:**

Cross-cultural adaptation process allowed us to prepare the final version of this tool, which was named NECPAL-BR. Collected data from pre-testing step enabled the analysis of semantic validity. The Content Validity Index observed at this step was 0.94.

**Conclusion:**

The semantic validity of the tool in its Portuguese version was confirmed; therefore, it may assist in screening chronic progressive disease patients, aiming to provide early palliative care. It may also be used to develop clinical and team performance indicators, and be employed as a care management tool designed to optimize resources.

## INTRODUCTION

Patient care in chronicity has been made evident in face of the demand for special care, which is modified throughout the health-disease process, initiating with measures of promotion and extending to palliative care.^(1)^ This modality of assistance is defined by the World Health Organization (WHO) as care delivered by a multiprofessional team, aiming to improve quality of life of patients and their families, facing a life-threatening disease, through prevention and relief of suffering, by means of early identification, impeccable assessment and treatment of pain and other physical, social, psychologic and spiritual symptoms.^(1)^

Patients with chronic diseases, especially when non-oncologic, are subject to care models strongly oriented towards acute conditions and events. Considering this bias, healthcare organizations have sought to develop policies of care based on a stratification of risks and needs, directing healthcare teams towards the appropriate use of technologies and medications, and supporting the change in paradigm.^(2)^

There is evidence that this strategy is an effective instrument for greater awareness given to health, causing positive impacts on clinical results, and increasing the efficiency of resource use.^(3)^

Among the measures for stratifying the demand for patient care during progression of chronic disease, this study focused on the identification of those who need to be included in the context of palliative care, in order to characterize both the signs of progressive worsening and the need to implement differentiated actions as early as possible – and not only in the advance phase of the disease.

As far as the authors know, there are no finalized studies in Brazil regarding the translated and validated instrument for identification of patients with need for palliative care that covers the different chronic and progressive diseases.

However, the *Institut Catala d’Oncologia* (ICO), in Spain, has used the NECPAL CCOMS-ICO^©^ instrument to identify patients requiring palliative care. This institute is one of the WHO collaborating centers assigned to develop a system of care to patients in palliative care. Such an instrument is a part of the *Proyecto* NECPAL CCOMS-ICO^©^ - *Identificación y Atención Integral-Integrada de Personas com Enfermedades Crónicas Avanzadas em Servicios de Salud y Sociales*, a project that has the essential purpose of improving palliative attention given to patients with early identification, expanding their activities to carriers of non-oncologic diseases and at any level of healthcare.^(4,5)^

Other researchers also developed guidelines, indicators, and instruments that aid in recognizing these patients, such as the Gold Standards Framework Prognostic Indicator Guidance (GSF PIG), the QUICK GUIDE to Identifying Patients for Supportive and Palliative Care, and the Supportive & Palliative Care Indicators Tool™ (SPCIT™).^(6-8)^ Although they have some similarities with the NECPAL CCOMS-ICO^©^, this one has the advantage of having been developed and applied in one of the WHO collaborating centers, whose results in palliative care are recognized as having a high impact, which justifies the choice of this instrument for translation and transcultural adaptation into Portuguese.

## OBJECTIVE

To perform the translation and transcultural adaptation of the NECPAL CCOMS-ICO^©^ instrument to Portuguese, and to analyze its semantic and content validity.

## METHODS

This is a methodological study of transcultural adaptation of the original version in Spanish of the NECPAL CCOMS-ICO^©^ instrument, translated into Portuguese, and of measuring its semantic and content validity in this language, carried out in the city of São Paulo, from 2016 to 2018. Authorization for translation and transcultural adaptation of NECPAL CCOMS-ICO^©^ into Portuguese was obtained by electronic contact with the authors of the instrument, who consented and made the survey in its original form available to us.

This project was approved by the Research Ethics Committees of the *Universidade Federal de São Paulo* (Unifesp), CAAE: 52850116.3.0000.5505, opinion 1.434.029, and of *Hospital Alemão Oswaldo Cruz*, CAAE: 52850116.3.3001.0070, opinion 1.456.900.

The transcultural adaptation of the NECPAL CCOMS-ICO^©^ instrument, based on the recommendations by Beaton,^(9)^ was performed as detailed in table 1.

**Table 1:** Transcultural adaptation of the instrument NECPAL CCOMS-ICO©

Stages	Actions
Translation	Performed by two independent translators who were native speakers of Portuguese and were fluent in Spanish, in which one of them was blinded to the study objectives (blind translation)
Synthesis of the translation	The investigators evaluated the translated versions to verify possible ambiguities or discrepancies in the translation process, and prepared a synthesis of the two versions
Back-translation	In this stage, the consensual version was back-translated into Spanish by two other translators, laypersons, with no clinical experience, blinded to the original instrument and to the study objectives; one of them was a native speaker of Spanish, besides having Portuguese proficiency
Committee of specialists	A committee of specialists analyzed all versions of the instrument as to semantic, idiomatic, conceptual, and cultural equivalence, based on the synthesis of the translations. A second round of evaluations was needed, which resulted in the pre-final version. At this stage, the content validity was also verified
Pre-test	Application of the pre-final version (n=35), in which the understanding of each item was assessed, that is, its semantic validity
Submission to the authors of the original text	Presentation of all reports to the authors of the original text. Approval of the final version

The specialist committee was composed as per Beaton’s recommendations.^(9)^Thus, four physicians and three nurses with more than two years of experience in care of patients with chronic progressive disease, one specialist in the Portuguese language, and one specialist in psychometric analysis, with a total of nine professionals. The group received all the versions of the NECPAL CCOMS-ICO^©^ by electronic means and recorded an evaluation of each item of the consensual instrument version in Portuguese, using a Likert scale, containing three affirmations: I fully agree, I partially agree, and I disagree.

After adjustment performed as per suggestions of the specialists, the instrument was forwarded to a second round of evaluations, for which a Likert scale was used containing three statements: essential; useful, but not essential; and unnecessary. Four physicians and five nurses were invited to participate in this committee. The proposal made to the group was 28 days for the feedback session, in addition to another 28 days for those who were unable to answer within this initial time. The data obtained in the judges’ analysis were used to verify the content validity (relevance) of the instrument.

Analysis of this psychometric measurement was made by agreement rate among the professionals, carried out by means of the Content Validity Ratio (CVR), which indicates the proportion according to the essential category relative to the total number of specialists, and then, the Content Validity Index (CVI), which indicates the mean CVR of all items, including those acceptable and those needing review. The minimum standard value for CVR is 0.58, and for CVI it is 0.70 to validate content of an instrument.^(10,11)^ For this study, we considered a minimum acceptable CVI of 0.80, as per the recommendations by Pasquali.^(10)^

At the end of the evaluation rounds by the committee of specialists, the investigators performed the suggested adjustments, resulting in the pre-final version, which was sent to the authors of the original instrument for evaluation of the translation and the transcultural adaptation. The authors made no changes. After receiving the approval of the authors of the original instrument, NECPAL CCOMS-ICO^©^ in its pre-final version was applied in a pilot test (pre-test) for analysis of the semantic validations (comprehension of the items).

The pre-test was conducted at inpatient units of a large private general hospital in the city of São Paulo. Patient recruitment was carried out at an adult inpatient unit, where primarily patients with medical diagnosis of chronic progressive disease are admitted, regardless of the etiology, sex, age group, and length of stay. The unit nurses, together with the investigator, collected data to identify those who could be invited to participate in the study. By convenience, seven patients with chronic progressive and irreversible diseases were selected, who accepted participation in the study and signed the patient’s Informed Consent Form (ICF).

Each patient was evaluated based on the pre-final version of NECPAL CCOMS-ICO^©^ by five professionals (physician, nurse, or psychologist) who agreed to participate in the study, had at least two years of experience in care of chronic patients, and signed the professional ICF. The total sample was made up of 35 professionals, as suggested in the literature for pre-test application.^(12)^ The healthcare professionals were considered the target population for the pre-test, since the evaluation to be done referred both to comprehension and acceptance of the instrument, as to its capacity to identify, by means of its indicators, chronic patients with progressive disease and requiring palliative care. The application of the pre-final version of the NECPAL CCOMS-ICO^©^ was done independently, based on the clinical evaluation of the evaluating professional and on the registrations made in the patient medical records. After the patient’s authorization, the professionals had five days to return the completed instrument to the investigators.

The data obtained in the pre-test were independently inserted into Excel spreadsheets, with independent double typing. After correction of errors and typing inconsistencies, the statistical analysis was done with the support of a professional. All tests were carried out with computational support of R, IBM (SPSS) version 21, and Excel 2010 (Microsoft Office) software. Data referring to the characterization of the research participants were analyzed by descriptive statistics, with categorical variables presented as absolute and relative frequency, and the quantitative variables as summary measures (mean, median, standard deviation, interquartile interval, and minimum and maximum values).

For analysis of the semantic validity, as in the previous stage, the agreement rate (CVR and CVI) among the professionals was calculated.

After analysis of the pre-test results, the modifications suggested in the pre-final version were made, originating the final version of this instrument.

## RESULTS

During the first stage of the transcultural adaptation process, comprised of translation of the original instrument NECPAL CCOMS-ICO^©^, from Spanish into Brazilian Portuguese, two versions of the instrument were created in Portuguese, T1 and T2, which were analyzed by the investigators, originating version T3. This version was back translated into Spanish, creating two versions, RT3a and RT3b. In reference to the harmonization of the items, the judges were questioned as to the existence of problems in grammatical structure, content fragmentation, confusing syntax, use of colloquial language, use of double negative induction, or if there was no apparent problem. Only five items of the instrument were pointed out by four or more judges (n=9) as having one or more of the stated problems.

After adaptation of T3, the pre-final version of the instrument was prepared. In the application of this pre-test version, we identified the item corresponding to the question “Demand: Has there been any implicit or explicit demand of limitation of therapeutic effort or demand for palliative care by a patient, the family, or team members?” presented with low agreement rate in its comprehension, that is, most professionals did not understand the item. Thus, aiming to maintain harmonization of the items, as to the translation into Portuguese, this item was described as “Demand: Has there been any explicit or implicit manifestation of limitation of therapeutic effort or request for palliative care on the part of the patient, the family, or team members?” The final version of the instrument NECPAL CCOMS-ICO^©^ was called NECPAL-BR (Appendix 1).

During the evaluation stage of the instrument in its T3 version by a committee of specialists in palliative care, it was possible to analyze the content validity of the NECPAL-BR instrument. To this end, the committee was questioned regarding the relevance of the items, using a Likert scale with three categories: essential; useful, but not essential; unnecessary.

For each item of the instrument, the CVR was calculated (Table 2). Since all the items obtained a CVR value greater than 0.58, there was no need to exclude any items. Therefore, considering the CVI of 0.87, the NECPAL CCOMS-ICO^©^ instrument in its Portuguese version showed content validity.

**Table 2:** Agreement rate among the professionals, according to the Content Validity Ratio, as to the relevance and comprehension of items of the instrument NECPAL CCOMS-ICO© in its Portuguese version

Item of the instrument	CVR as to relevance (content validity)	CVR as to comprehension (semantic validity)
1. Surprise question	0.94	0.91
2.1. Demand	1.00	0.74
2.2. Necessity	0.97	0.89
3.1. Nutritional decline	1.00	1.00
3.2.Functional decline	1.00	0.94
3.3. Cognitive decline	0.97	0.91
4. Severe dependence	1.00	0.97
5. Geriatric syndromes	1.00	1.00
6. Persistent symptoms	0.97	1.00
7.1. Emotional suffering or severe adaptive disorder	1.00	0.91
7.2. Severe social vulnerability	0.66	0.91
8. Multimorbidity	1.00	1.00
9. Use of resources	0.94	1.00
10. Specific indicators	1.00	1.00

CVR: content validity ratio.

The semantic validity was verified in the pre-test. The sample was composed of 35 professionals, most of them nurses, age range of 24-56 years, who had at least 8.5 years of experience (standard deviation=6.9). As to training in palliative care, only five professionals had been trained in this field.

For each item of the instrument under evaluation, the comprehension was questioned. The evaluator was to indicate if the item was easily understood; not easy nor difficult to understand; or difficult to understand. Thus, the CVR of each item was calculated, that is, the proportion of agreement with the “easily understood” category relative to the number of specialists, which also can be observed in table 2. Next, the CVI of 0.94 was obtained, that is, the instrument presented with semantic validity.

## DISCUSSION

One main evidence of this study was the identification of the content validity by means of CVI of 0.87, since this is an important phase of the development and adaptation of questionnaires and scales. However, the original study does not specify content validity test values. The authors of that study reported that such a psychometric measure was evaluated by a logical-rational process, of the clarity and acceptability of the instrument, considering individual interviews structured as the convenience sample of 18 professionals (physicians, nurses, and psychologists) of services with a high prevalence of chronic patients. They also asserted that, as a result, the instrument offers a guarantee of content validity when compared to the GSF PIG, in reference to semantic, idiomatic, experiential, and conceptual equivalence.^(5)^

The second aspect of great relevance was the semantic validity, presenting a CVI of 0.94, which is a very expressive result. Nevertheless, there were no conditions to compare it with the original instrument, since there is no register of the performance of this test by the Spanish authors.^(5)^We point out that is only the beginning of a process, both for the authors of the original instrument and for the investigators of this study, which should also include other tests to evaluate the remaining psychometric measures relevant to such instrument.

A secondary finding may be observed in the semantic validity test when calculating the CVR of the question “Demand: Has there been any implicit or explicit demand of limitation of therapeutic effort or demand for palliative care on the part of the patient, the family, or team members?” that appears as the item with the lowest CVR (0.74). Considering it is a single datum with a value lower that the others of the instrument, and even so, above the borderline value, which would require a revision of the item, after discussion of the investigators with the statistician, the choice was made to modify the description of the question, without submitting it to a new round of evaluation. The investigators believed that the word “demand,” utilized more than once in the phrase with different meanings, might have been the reason for less understanding of the item. Therefore, this word was replaced by its synonyms in the new wording of the question.

This instrument still has one point of attention. It is the emotional status evaluation scale (Emotional Malaise Detection), which was provided to the participants in free translation, but would need a validated transcultural adaptation, so that it could be completed by the professional in a non-subjective mode.

Despite the limitations pointed out, this instrument is beneficial for clinical practice. Among the benefits, we highlight that it can help in the triage of patients who experience a chronic progressive disease, with a view to offering early appropriate palliative care. Additionally, it can enable the development of clinical, team performance and management indicators, for example, and be used as a tool for care management and optimization of resources. Another aspect that can be driven by using NECPAL-BR is the investigative practice, enabling the creation of institutional protocols and affording evidence-based practice, focused on excellence. Cultural change and institutional support are necessary in this modality of care to make these benefits feasible.

The authors of the original instrument conducted a study evaluating the (bio)ethical implications related to the early identification of patients with this advanced chronic disease. The discussions were held by an ethics committee with specialists from this field of care, and generally concluded that early identification, coupled with delivery of differentiated care required as a result, provides substantial quality of palliative care for patients with advanced chronic diseases. The authors pointed out barriers against early identification - denial of healthcare professionals (“we have already been doing that”, “there are many needy patients”), increased workloads, lack of training to meet the needs of patients and their families, and corporative resistance for the practice of integrated care.^(13)^

The authors reinforced as potential benefits the fact that early identification is a new perspective both for patients (and families), and for professionals and services; it generates a reflexive process about your care needs and objectives; it promotes a gradual expansion of the palliative approach, as well as increased autonomy by means of anticipated care planning; it facilitates a rational and thoughtful decision-making process; instigates active discussion and therapeutic goal revision; promotes integrated and continuous care; and allows a rational approach to emergency care.^(13)^

Early identification of all types of chronic patients with palliative care needs and limited life prognosis in the health services is one of the most relevant recent challenges of palliative care policies. Thus, the implementation of systemic policies for the early identification of palliative needs, at the expense of conventional needs or concomitant with them, should be encouraged and accompanied by a model of care that contains actions based on consensus among specialists, and training programs that employ professionals to care for patients with maximum benefits for them.

## CONCLUSION

In this study, the translation, adaptation and semantic validation in Brazil of the NECPAL CCOMS-ICO^©^ instrument were carried out. During the first stage of the transcultural adaptation process, five versions of the instrument were generated, until reaching the final version of the instrument, called NECPAL-BR.

The instrument has understandable and relevant items, that is, there was agreement between the requested capacity in a specific domain and the performance requested in the test, which deals with measuring that domain, having content and semantic validity in its Portuguese version.

NECPAL-BR should be able to be submitted to other psychometric tests in a later study, being implemented in clinical practice, and as a comparative element in other investigations related to the identification of patients with chronic progressive diseases.

## Appendix 1

**Table:** NECPAL-BR instrument

Palliative needs			
An instrument for the identification of people with advanced and/or end-stage diseases and the need for palliative care for use in health and social services			
**Surprise question**	Would you be surprised if this patient died over the next year?		[ ] Yes [ ] No
**Demand or need**	Demand: has there been any explicit or implicit manifestation of limitation of therapeutic effort, or request for palliative care by the patient, their family or team members?		[ ] Yes [ ] No
	Need: identified by health team professionals		[ ] Yes [ ] No
**General clinical indicators in the last 6 months** - Severe, persistent, progressive, not related to recent intercurrent process	Nutritional decline	Weight loss >10%	[ ] Yes [ ] No
- Combine severity WITH progression	Functional decline	Worsening of Karnofsky or Barthel> 30%	[ ] Yes [ ] No
		Loss of more than two ADL	
	Cognitive decline	Mini Mental Loss ≥5 or Pfeiffer ≥3	[ ] Yes [ ] No
**Severe dependency**	Karnofsky<50 or Barthel<20		[ ] Yes [ ] No
**Geriatric syndromes**	Pressure lesion	Recurrent or persistent clinical data from the medical history ≥2	[ ] Yes [ ] No
	Repeat infections		
	Delirium		
	Dysphagia		
	Falls		
**Persistent symptoms**	Pain, tiredness, nausea, depression, anxiety, sleepiness, lack of appetite, malaise, dyspnea, and insomnia	≥2 recurring or persistent ESAS symptoms	[ ] Yes [ ] No
**Psychosocial aspects**	Emotional distress or severe adaptive disorder	Detection of Emotional Discomfort >9	[ ] Yes [ ] No
	Severe social vulnerability	Social and family evaluation	[ ] Yes [ ] No
**Multimorbidity**	≥2 advanced chronic diseases or conditions (from the attached list of specific indicators)		[ ] Yes [ ] No
**Use of resources**	Evaluation of the demand or intensity of interventions	More than two urgent admissions (unscheduled) in 6 months Increased demand for or intensity of interventions (home care and nursing interventions)	[ ] Yes [ ] No
**Specific indicators**	Cancer, COPD, CHF, liver failure, kidney failure, stroke, dementia, neurodegenerative diseases, AIDS, and other advanced diseases	In Appendix 2: evaluation of the criteria of severity and progression	[ ] Yes [ ] No

Translated from: Gómez-Batiste X, Martínez-Muñoz M, Blay C, Amblàs J, Vila L, Costa X, et al. Instrumento NECPAL CCOMS-ICO©: identificación de pacientes com enfermedades crónicas evolutivas y necesidades de atenciones y medidas paliativas en servicios de salud y sociales. Centro Colaborador de la OMS para Programas Públicos de Cuidados Paliativos [Internet]. Institut Català d’Oncologia 2011 [cited 2018 May 25]. Available from: http://ico.gencat.cat/web/.content/minisite/ico/professionals/documents/qualy/arxius/doc_necpal_ccoms-ico_instrumento_doc_generalv1_esp_vf_201203.pdf [Translation authorized by the author]. ADL: activities of daily living; ESAS: Edmonton Symptom Evaluation Scale; COPD: chronic obstructive pulmonary disease; CHF: congestive heart failure.

## Appendix 2

**Table:** Specific indicators

NECPAL criteria of severity/progression/advanced disease*	
**Oncologic disease**	Metastatic or advanced locoregional cancer
	In progression in solid tumors
	Persistent, poorly controlled, or refractory symptoms, despite optimization of the specific treatment
**Chronic pulmonary disease**	Dyspnea at rest and upon minimal exertion between decompensations
	Restricted to home with walking limitations
	Spirometric criteria of severe obstruction (FEV1 <30%) or criteria of severe restrictive deficit (FVC <40%/DLCO <40%)
	Baseline gasometric criteria of continuous home oxygen therapy
	Need for continuous corticotherapy
	Associated symptomatic heart failure
**Chronic heart disease**	Dyspnea at rest or upon minimal exertion between the decompensations
	NYHA classes III or IV heart failure, severe non-surgical valvar disease or non-revascularizable coronary artery disease
	Baseline echocardiogram: EF <30% or serious PH (PASP >60)
	Associated renal failure (GFR<60mL/min/1.73m^2^)
	Association with renal failure and persistent hyponatremia
**Dementia**	GDS≥6c
	Progression of cognitive, functional, and/or nutritional decline
**Fragility**	CSHA Fragility Index ≥0.5
	Comprehensive geriatric evaluation suggestive of advanced fragility
**Vascular neurological disease (stroke)**	During the acute and subacute phases (<3 months after stroke): persistent vegetative status or minimal consciousness >3 days
	During the chronic phase (>3 months after stroke): repeated medical complications (or dementia with seriousness criteria after stroke)
**Degenerative neurologic diseases: ALS, multiple sclerosis, and Parkinson’s disease**	Progressive worsening of the physical and/or cognitive function
	Complex and difficult to control symptoms
	Persistent dysphagia
	Persistent speech disorder
	Increasing difficulties in communication
	Recurrent pneumonia due to aspiration, dyspnea, or respiratory failure
**Chronic hepatic disease**	Advanced cirrhosis (Child C stage) (determined in patient with no complications, or treated complications, and optimized treatment), MELD-Na>30 or refractory ascites, hepatorrenal syndrome, or upper digestive hemorrhage due to persistent portal hypertension despite optimized treatment Presence of hepatocellular carcinoma stage C or D
**Severe chronic renal disease**	Severe renal failure (GFR<15mL/minute) in patients who are not candidates or who refuse replacement treatment and/or transplant Finalization of dialysis or failure of the transplant

* Use validated instruments for severity and/or prognosis based on experience and evidence; in all cases, also assess emotional suffering or severe functional impact on patients (and/or impact on the family) such as criteria for palliative needs; in all cases, evaluate ethical dilemmas in decision making; always evaluate the combination with multiple conditions. FEV1: maximum expiratory volume in 1 second; FVC: forced vital capacity; DLCO: diffusing capacity of carbon monoxide; NYHA: New York Heart Association; EF: ejection fraction; PH: Pulmonary hypertension; PASP: pulmonary artery systolic pressure; GFR: glomerular filtration rate; GDS: Geriatric Depression Scale; CSHA: Canadian Study of Health and Aging; ALS: Amyotrophic lateral sclerosis; MELD-Na: Model for End-Stage Liver Disease-Sodium.

**Table:**

Classification	
Surprise question	Surprise question + (Would not surprise me)
	Surprise question – (Would surprise me)
NECPAL Parameters	NECPAL + (from 1 to 13 “Yes” answers)
	NECPAL – (no highlighted parameter)
**Encoding and registry**	Propose encoding, such as Patient with Advanced Chronicity if Surprise question + and NECPAL +
