# Supplementary material for: Cross-cultural adaptation and semantic validation of an instrument to identify palliative requirements in Portuguese
Source: Einstein (Sao Paulo). 2020 Oct 2;18:eAO5539. doi: 10.31744/einstein_journal/2020AO5539 (PMC7531898; doi:10.31744/einstein_journal/2020AO5539)
Supplement: Translation

# Translation: Adaptação transcultural e validação semântica de instrumento para identificação de necessidades paliativas em língua portuguesa

**Authors:** Marcella Tardeli Esteves Angioleti Santana, Xavier Gómez-Batiste, Lucia Marta Giunta da Silva, Maria Gaby Rivero de Gutiérrez

**Affiliations:** 1 Brasil Universidade Federal de São Paulo, São Paulo, SP, Brasil.; 2 Spain Institut Català d’Oncologia, Barcelona, Spain.

**Keywords:** Cuidados paliativos, Doença crônica, Sistemas de identificação de pacientes, Administração dos cuidados ao paciente, Inquéritos e questionários, Estudo de validação

## Abstract

**Objetivo:**

Realizar a tradução e a adaptação transcultural, e analisar a validade semântica do instrumento NECPAL CCOMS-ICO^©^ para a língua portuguesa.

**Métodos:**

Pesquisa metodológica, de adaptação transcultural da versão espanhola do instrumento para a língua portuguesa e mensuração da validade semântica. Conduziu-se a adaptação transcultural com base nas recomendações de Beaton, que inclui tradução, síntese da tradução, retrotradução, e análise da equivalência semântica, idiomática, conceitual e cultural das versões, resultando na versão pré-final, a qual foi submetida ao pré-teste (n=35). Para analisar a validade semântica, foi calculado o Índice de Validade de Conteúdo.

**Resultados:**

O processo de adaptação transcultural possibilitou a elaboração da versão final, denominada NECPAL-BR. Os dados do pré-teste possibilitaram a análise da validade semântica. O Índice de Validade de Conteúdo obtido nessa etapa foi de 0,94.

**Conclusão:**

O instrumento possui validade semântica em sua versão em língua portuguesa e, portanto, pode auxiliar na triagem de pacientes com doença crônica progressiva, com vistas a oferecer, de forma precoce, atenção paliativa. Pode, ainda, propiciar o desenvolvimento de indicadores clínicos, de desempenho de equipe e servir como ferramenta de gestão do cuidado, visando à otimização de recursos.

## INTRODUÇÃO

O cuidado na cronicidade tem se evidenciado diante da demanda de cuidados especiais, que se modificam ao longo do processo saúde-doença, iniciando-se com medidas de promoção e se estendendo até os cuidados paliativos.^(1)^ Esta modalidade de assistência é definida pela *World Health Organization* (WHO) como o cuidado promovido por uma equipe multidisciplinar, que objetiva a melhoria da qualidade de vida do paciente e seus familiares, diante de uma doença que ameace a vida, por meio da prevenção e alívio do sofrimento, da identificação precoce, da avaliação impecável e do tratamento da dor e demais sintomas físicos, sociais, psicológicos e espirituais.^(1)^

Pacientes com doenças crônicas, principalmente as não oncológicas, estão sujeitos a modelos de atenção à saúde fortemente orientados para condições e eventos agudos. Diante desse viés, as organizações de saúde têm buscado o desenvolvimento de políticas de cuidado que funcionem a partir da estratificação de riscos e necessidades, direcionando as equipes de saúde para o uso apropriado de tecnologias e medicamentos, e apoiando a mudança de paradigma.^(2)^

Há evidências de que essa estratégia constitui instrumento efetivo para prestar melhor atenção à saúde, causar impactos positivos nos resultados clínicos e aumentar a eficiência no uso dos recursos.^(3)^

Dentre as medidas para estratificar a demanda de cuidado de pacientes em progressão da doença crônica, este estudo focou na identificação daqueles que necessitam ser incluídos no contexto dos cuidados paliativos, a fim de caracterizar tanto os sinais de piora progressiva quanto a necessidade de implementar ações diferenciadas o mais precocemente possível – e não apenas na fase avançada da doença.

Não é do conhecimento dos autores a existência de estudos já finalizados, no Brasil, sobre instrumento traduzido e validado para identificação de pacientes com necessidade de cuidados paliativos que englobem as diferentes condições e patologias crônicas progressivas.

No entanto, percebeu-se que o *Institut Catala d’Oncologia* (ICO), na Espanha, tem utilizado o instrumento NECPAL CCOMS-ICO^©^ para identificar pacientes com necessidade de atenção paliativa. Este instituto é um dos centros colaboradores designados pela WHO para desenvolver um sistema de atendimento aos pacientes em cuidados paliativos. Tal instrumento faz parte do *Proyecto* NECPAL CCOMS-ICO^©^ - *Identificación y Atención Integral-Integrada de Personas com Enfermedades Crónicas Avanzadas em Servicios de Salud y Sociales*, um projeto que tem como propósito fundamental melhorar a atenção paliativa mediante a identificação precoce desses pacientes, expandindo sua atuação para portadores de doenças não oncológicas e em qualquer nível de atendimento em saúde.^(4,5)^

Outros pesquisadores também desenvolveram guias, indicadores e instrumentos que auxiliam no reconhecimento desses pacientes, como o *Gold Standards Framework Prognostic Indicator Guidance* (GSF PIG), o *QUICK GUIDE to Identifying Patients for Supportive and Palliative Care*, o *Supportive & Palliative Care Indicators Tool*™ (SPCIT™).^(6-8)^ Embora tenham algumas similaridades com o NECPAL CCOMS-ICO^©^, este tem a vantagem de ter sido desenvolvido e aplicado em um dos centros colaboradores da WHO, cujos resultados na atenção paliativa são reconhecidos como de alto impacto, o que justifica a escolha deste instrumento para tradução e adaptação transcultural para a língua portuguesa.

## OBJETIVO

Realizar a tradução e adaptação transcultural do instrumento NECPAL CCOMS-ICO^©^ para a língua portuguesa e analisar a sua validade semântica e de conteúdo.

## MÉTODOS

Trata-se de um estudo metodológico de adaptação transcultural da versão original em espanhol do instrumento NECPAL CCOMS-ICO^©^ para a língua portuguesa e mensuração da validade semântica e de conteúdo nesse idioma, realizado em São Paulo, do ano de 2016 a 2018. A autorização para tradução e adaptação transcultural do NECPAL CCOMS-ICO^©^ para a língua portuguesa foi obtida mediante contato eletrônico com os autores do instrumento, os quais consentiram e disponibilizaram o inventário em sua forma original.

Este projeto foi aprovado pelos Comitês de Ética em Pesquisa da Universidade Federal de São Paulo (Unifesp), CAAE: 52850116.3.0000.5505, parecer 1.434.029, e do Hospital Alemão Oswaldo Cruz, CAAE: 52850116.3.3001.0070, parecer 1.456.900.

A adaptação transcultural do instrumento NECPAL CCOMS-ICO^©^, conduzida com base nas recomendações de Beaton,^(9)^ deu-se conforme detalhado na tabela 1.

**Tabela 1:** Adaptação transcultural do instrumento NECPAL CCOMS-ICO©

Etapas	Ações
Tradução	Realizada por dois tradutores independentes, que tinham o português como língua nativa e com domínio do espanhol, sendo que um deles desconhecia os objetivos do estudo (tradução cega)
Síntese da tradução	Os pesquisadores avaliaram as versões traduzidas para verificar possíveis ambiguidades ou discrepâncias no processo de tradução e elaboraram a síntese das duas versões
Retrotradução	Nesta etapa, a versão consensual foi vertida para o espanhol, por outros dois tradutores diferentes, leigos, sem experiência clínica, desconhecedores do instrumento original e dos objetivos do estudo, sendo que um deles tinha o espanhol como língua nativa, além de domínio do português
Comitê de especialistas	Um comitê de especialistas analisou todas as versões do instrumento quanto à equivalência semântica, idiomática, conceitual e cultural, usando a síntese das traduções como base. Uma segunda rodada de avaliação se fez necessária, o que resultou na versão pré-final. Nesta etapa, foi também verificada a validade de conteúdo
Pré-teste	Aplicação da versão pré-final (n=35), na qual foi avaliado o entendimento de cada item, ou seja, sua validade semântica
Submissão aos autores do original	Apresentação de todos os relatórios aos autores do original. Aprovação da versão final

A composição do comitê de especialistas também se deu com base nas recomendações de Beaton.^(9)^ Assim, foram convidados quatro médicos e três enfermeiros que tinham mais de dois anos de experiência no cuidado com pacientes com doença crônica progressiva, um especialista em língua portuguesa e um especialista em análise psicométrica, totalizando nove profissionais. O grupo recebeu todas as versões do NECPAL CCOMS-ICO^©^, por meio eletrônico, e registrou sua avaliação de cada item do instrumento na versão consensual em português, utilizando uma escala do tipo Likert, contendo três afirmações: concordo plenamente, concordo parcialmente e discordo.

Após ajustes realizados conforme sugestões dos especialistas, o instrumento foi encaminhado a uma segunda rodada de avaliação, para a qual foi utilizada uma escala do tipo Likert, contendo três afirmações: essencial; útil, mas não essencial; e desnecessário. Foram convidados a participar deste comitê quatro médicos e cinco enfermeiros. Foi proposto ao grupo um prazo de 28 dias para a devolutiva, acrescidos de mais 28 dias para aqueles que não conseguiram responder neste prazo inicial. Os dados obtidos na análise dos juízes foram utilizados para verificar da validade de conteúdo (pertinência) do instrumento.

A análise dessa medida psicométrica deu-se pelo índice de concordância entre os profissionais, realizado por meio da *Content Validity Ratio* (CVR), que indica a proporção de acordo na categoria essencial em relação ao número total de especialistas e, em seguida, o Índice de Validade de Conteúdo (IVC), que indica a média da CVR de todos os itens, incluindo os aceitáveis e os que precisariam de revisão. O padrão mínimo aceitável da CVR é de 0,58 e do IVC é de 0,70 para a validade de conteúdo de um instrumento.^(10,11)^ Para este estudo, considerou-se IVC mínimo aceitável de 0,80, seguindo as recomendações de Pasquali.^(10)^

Ao término das rodadas de avaliação pelo comitê de especialistas, os pesquisadores realizaram os ajustes sugeridos, resultando na versão pré-final, que foi enviada aos autores do instrumento original, para avaliação da tradução e da adaptação transcultural realizada. Os autores não fizeram nenhuma alteração. Após recebimento da anuência dos autores do instrumento original, o NECPAL CCOMS-ICO^©^ em sua versão pré-final foi aplicado em um teste piloto (pré-teste) para análise da validação semântica (compreensão dos itens).

O pré-teste foi realizado em unidades de internação de um hospital geral particular, de grande porte, em São Paulo. O recrutamento dos pacientes foi realizado em uma unidade de internação de adultos, na qual são alocados principalmente pacientes com diagnóstico médico de doença crônica em progressão, independente da etiologia de base, sexo, faixa etária e tempo de internação. A coleta de dados contou com auxílio dos enfermeiros das unidades, em conjunto com a pesquisadora, para identificação daqueles que pudessem ser convidados a participarem do estudo. Foram selecionados, por conveniência, sete pacientes com doenças crônicas progressivas e irreversíveis que aceitaram participar do estudo e assinaram o Termo de Consentimento Livre e Esclarecido (TCLE) do paciente.

Cada paciente foi avaliado com base na versão pré-final do NECPAL CCOMS-ICO^©^ por cinco profissionais (médico, enfermeiro ou psicólogo), que aceitaram participar do estudo, possuíam, no mínimo, 2 anos de experiência na assistência a pacientes crônicos e assinaram o TCLE do profissional. A amostra totalizou 35 profissionais, conforme sugere a literatura para realização do pré-teste.^(12)^ Os profissionais de saúde foram considerados população-meta para realização do pré-teste, posto que a avaliação a ser realizada referia-se tanto à compreensão e aceitação do instrumento, quanto à sua capacidade para identificar, por meio dos indicadores nele contidos, pacientes crônicos em fase de progressão da doença, que demandassem atenção paliativa. A aplicação da versão pré-final do NECPAL CCOMS-ICO^©^ foi feita de forma independente, com base na avaliação clínica do profissional avaliador e nos registros dos prontuários dos pacientes. Após a autorização do paciente, os profissionais tiveram cinco dias para devolver aos pesquisadores o instrumento preenchido.

Os dados obtidos no pré-teste foram inseridos em planilhas do programa Excel, com dupla digitação independente. Após a correção de erros e inconsistências da digitação, foi realizada a análise estatística, com apoio de um profissional da área. Todos os testes foram realizados com apoio computacional dos softwares R, IBM (SPSS), versão 21, e Excel 2010 (Microsoft Office). Os dados referentes à caracterização dos participantes da pesquisa foram analisados por estatística descritiva, sendo as variáveis categóricas apresentadas sob forma de frequência absoluta e relativa e as quantitativas, como medidas-resumo (média, mediana, desvio padrão, intervalo interquartil, e valores mínimo e máximo).

Para a análise da validade semântica, como na etapa anterior, calculou-se o índice de concordância entre os profissionais (CVR e IVC).

Após a análise dos resultados do pré-teste, foram realizadas as alterações sugeridas na versão pré-final, originando a versão final deste instrumento.

## RESULTADOS

Na primeira etapa do processo de adaptação transcultural, constituída pela tradução do instrumento original em espanhol NECPAL CCOMS-ICO^©^ para a língua portuguesa do Brasil, foram geradas duas versões do instrumento em português, T1 e T2, as quais foram analisadas pelos pesquisadores, dando origem à versão T3. Esta versão foi vertida para o espanhol, gerando duas versões: RT3a e RT3b. No que se referiu à harmonização dos itens, foi questionado aos juízes se existiam problemas na estrutura gramatical, na fragmentação do conteúdo, na sintaxe confusa, no uso de linguagem coloquial, na dupla negativa/ indução ou se não havia problema aparente. Apenas cinco itens do instrumento foram apontados por quatro ou mais juízes (n=9) como tendo um ou mais dos problemas citados.

Após adequação da T3, elaborou-se a versão pré-final do instrumento. Na aplicação desta versão no pré-teste, identificou-se que o item que correspondia à pergunta “Demanda: Tem havido alguma demanda implícita ou explícita de limitação de esforço terapêutico ou demanda de atenção paliativa por parte do(a) paciente, família ou membros da equipe?” apresentou baixa concordância sobre sua compreensão, ou seja, a maioria dos profissionais não compreendeu o item. Assim, visando manter a harmonização dos itens, no que se referia à tradução para a língua portuguesa, este item ficou descrito como “Demanda: Tem havido alguma manifestação explícita ou implícita, de limitação de esforço terapêutico ou pedido de atenção paliativa por parte do(a) paciente, sua família ou membros da equipe?”. A versão final do instrumento NECPAL CCOMS-ICO^©^ foi, então, nomeada NECPAL-BR (Apêndice 1).

Durante a etapa de avaliação do instrumento em sua versão T3 por um comitê de especialistas em cuidados paliativos, foi possível analisar a validade de conteúdo do instrumento NECPAL-BR. Para tanto, o comitê foi questionado a respeito da pertinência dos itens, utilizando uma escala do tipo Likert com três categorias: essencial; útil, porém não essencial; e desnecessário.

Para cada um dos itens do instrumento, calculou-se a CVR (Tabela 2). Como todos os itens obtiveram CVR maior que 0,58, não houve necessidade de exclusão de algum item. Assim, considerando o IVC de 0,87, o instrumento NECPAL CCOMS-ICO^©^ em sua versão em língua portuguesa apresentou validade de conteúdo.

**Tabela 2:** Índice de concordância entre os profissionais, conforme a *Content Validity Ratio*, sobre a pertinência e a compreensão dos itens do instrumento NECPAL CCOMS-ICO© na sua versão em língua portuguesa

Item do instrumento	CVR sobre pertinência (validade de conteúdo)	CVR sobre compreensão (validade semântica)
1. Pergunta surpresa	0,94	0,91
2.1. Demanda	1,00	0,74
2.2. Necessidade	0,97	0,89
3.1. Declínio nutricional	1,00	1,00
3.2. Declínio funcional	1,00	0,94
3.3. Declínio cognitivo	0,97	0,91
4. Dependência grave	1,00	0,97
5. Síndromes geriátricas	1,00	1,00
6. Sintomas persistentes	0,97	1,00
7.1. Sofrimento emocional ou transtorno adaptativo grave	1,00	0,91
7.2. Vulnerabilidade social grave	0,66	0,91
8. Multimorbidade	1,00	1,00
9. Uso de recursos	0,94	1,00
10. Indicadores específicos	1,00	1,00

CVR: *content validity ratio*.

A validade semântica foi averiguada no pré-teste. A amostra foi composta por 35 profissionais, em sua maioria enfermeiros, com idade que variou entre 24 e 56 anos e que tinham, em média, 8,5 anos de tempo de experiência (desvio padrão=6,9). No que se refere à formação em cuidados paliativos, apenas cinco profissionais possuíam formação nessa área.

Para cada item do instrumento em avaliação, questionou-se a compreensão. O avaliador deveria indicar se o item era: de fácil compreensão; nem fácil, nem difícil compreensão; ou de difícil compreensão. Assim, calculou-se a CVR de cada item, ou seja, a proporção de acordo com a categoria “fácil compreensão” em relação ao número de especialistas, que também pode ser observada na tabela 2. Obteve-se, em seguida, IVC de 0,94, ou seja, o instrumento apresentou validade semântica.

## DISCUSSÃO

Uma das principais evidências deste estudo foi a constatação da validade de conteúdo, por meio do IVC de 0,87, já que se trata de uma fase importante do desenvolvimento e da adaptação de questionários e escalas. O estudo original, entretanto, não explicita valores de testes de validade de conteúdo. Os autores daquele estudo referem que tal medida psicométrica foi avaliada por um processo lógico-racional, da clareza e da aceitabilidade do instrumento, mediante entrevistas individuais estruturadas com amostra de conveniência de 18 profissionais (médicos, enfermeiros e psicólogos) de serviços com alta prevalência de pacientes crônicos. Afirmam também que, como resultado, o instrumento oferece garantia de validade de conteúdo, comparado ao GSF PIG, em relação à equivalência semântica, idiomática, vivencial e conceitual.^(5)^

O segundo aspecto de grande relevância foi a validade semântica, que apresentou IVC de 0,94, que consiste em resultado bastante expressivo. Não houve, porém, condições de compará-lo ao índice do instrumento original, visto que não há registro da realização desse teste pelos autores espanhóis.^(5)^ Cabe ressaltar que esse é somente o início de um processo, tanto para os autores do instrumento original como para os pesquisadores deste estudo, que deve englobar também outros testes para avaliar as demais medidas psicométricas pertinentes a tal instrumento.

Um achado secundário pode ser observado no teste de validade semântica, ao se calcular a CVR da pergunta “Demanda: Tem havido alguma demanda implícita ou explícita de limitação de esforço terapêutico ou demanda de atenção paliativa por parte do(a) paciente, família ou membros da equipe?”, que aparece como o item de CVR mais baixa (0,74). Por ser um dado único com valor inferior em relação aos demais do instrumento e, ainda assim, acima do valor limítrofe, que exigiria uma revisão do item, após discussão das pesquisadoras com o estatístico, optou-se por modificar a descrição da pergunta, sem submetê-la à nova rodada de avaliação. As pesquisadoras acreditaram que a palavra “demanda”, utilizada mais de uma vez na frase com sentidos diferentes, pode ter sido a razão da menor compreensão do item. Desse modo, essa palavra foi substituída por seus sinônimos na nova grafia da pergunta.

Este instrumento ainda possui um ponto de atenção. Trata-se da escala de avaliação do estado emocional (Detecção do Mal-Estar Emocional), que foi concedida aos participantes em tradução livre, mas que precisaria de uma adaptação transcultural validada, para que esse item pudesse ser preenchido pelo profissional de modo não subjetivo.

Apesar das limitações apontadas, esse instrumento traz benefícios para a prática clínica. Dentre as quais, destaca-se que pode auxiliar na triagem de pacientes que vivenciam uma doença crônica progressiva, com vistas a oferecer, de forma precoce, atenção paliativa apropriada. Pode, ainda, propiciar o desenvolvimento de indicadores clínicos, de desempenho de equipe e de gerenciamento, por exemplo, e servir como ferramenta de gestão do cuidado e de otimização de recursos. Outro aspecto que pode ser impulsionado pelo uso do NECPAL-BR é a prática investigativa, possibilitando a criação de protocolos institucionais e proporcionando prática baseada em evidências, com foco na excelência. Para que esses benefícios sejam factíveis, é necessária uma mudança cultural e apoio institucional, no que se refere a essa modalidade do cuidado.

Os autores do instrumento original desenvolveram um estudo que avalia as implicações (bio)éticas relacionadas à identificação precoce dos pacientes com doença crônica avançada. As discussões foram realizadas por um comitê de ética junto de especialistas nesta área de cuidado e, de modo geral, concluíram que a identificação precoce, associada à prestação do cuidado diferenciado que se exige por consequência, proporciona qualidade de atenção paliativa substancial aos pacientes com doenças crônicas avançadas. Foram apontadas pelos autores, como barreiras para a identificação precoce, a negação de profissionais de saúde (“já estamos fazendo isso”, “há muitos pacientes necessitados”), o aumento da carga de trabalho, a falta de treinamento para atender às necessidades dos pacientes e das famílias, e as resistências corporativas para a prática do cuidado integrado.^(13)^

Os autores reforçam como potenciais benefícios o fato de que a identificação precoce é, tanto para pacientes (e famílias), quanto para profissionais e serviços, uma nova perspectiva; gera um processo reflexivo sobre suas necessidades e objetivos de cuidado; promove a ampliação gradual da abordagem paliativa e um aumento da autonomia por meio do planejamento antecipado de cuidados; facilita um processo racional e reflexivo de tomada de decisão; instiga discussão ativa e revisão de metas terapêuticas; promove cuidados integrados e contínuos; e permite uma abordagem racional ao atendimento de emergência.^(13)^

A identificação precoce de todos os tipos de pacientes crônicos com necessidades de cuidados paliativos e prognóstico de vida limitado nos serviços de saúde é um dos desafios recentes mais relevantes das políticas de cuidados paliativos. Assim, a implementação de políticas sistêmicas para a identificação precoce de necessidades paliativas, em detrimento das necessidades convencionais ou em concomitância a elas, deve ser incentivada, bem como vir acompanhada de um modelo de atenção que contenha ações baseadas no consenso entre especialistas e de programas de treinamento que instrumentalizem os profissionais para o atendimento dos pacientes com o máximo de benefícios para eles.

## CONCLUSÃO

Neste estudo, desenvolveram-se a tradução, a adaptação e a validação semântica no Brasil do instrumento NECPAL CCOMS-ICO^©^. Na primeira etapa do processo de adaptação transcultural, foram geradas cinco versões do instrumento, até que se chegasse à versão final do instrumento, denominada NECPAL-BR.

O instrumento tem itens compreensíveis e pertinentes, ou seja, houve concordância entre a capacidade solicitada em um domínio específico e o desempenho solicitado no teste, que trata de medir esse domínio, possuindo validade de conteúdo e semântica em sua versão em língua portuguesa.

O NECPAL-BR deve poder ser submetido a demais testes psicométricos em um estudo posterior, sendo implantado na prática clínica e como elemento comparativo em outras pesquisas relacionadas à identificação de pacientes com doenças crônicas progressivas.

## Apêndice 1

**Table:** Instrumento NECPAL-BR

Necessidades paliativas			
Instrumento para identificação de pessoas em situação de doença avançada e/ou terminais e necessidade de atenção paliativa para uso em serviços de saúde e sociais			
Pergunta surpresa	Você ficaria surpreso se este paciente morresse ao longo do próximo ano?		[ ] Sim [ ] Não
Demanda ou necessidade	Demanda: tem havido alguma manifestação explícita ou implícita, de limitação de esforço terapêutico ou pedido de atenção paliativa por parte do(a) paciente, sua família ou membros da equipe?		[ ] Sim [ ] Não
	Necessidade: identificada por profissionais da equipe de saúde		[ ] Sim [ ] Não
**Indicadores clínicos gerais nos últimos 6 meses** - Graves, persistentes, progressivos, não relacionados com processo intercorrente recente - Combinar gravidade COM progressão	Declínio nutricional	Perda de peso >10%	[ ] Sim [ ] Não
	Declínio funcional	Piora do Karnofsky ou Barthel >30%	[ ] Sim [ ] Não
	Declínio cognitivo	Perda de mais que duas ABVDs	[ ] Sim [ ] Não
		Perda ≥5 Minimental ou ≥3 Pfeiffer	
**Dependência grave**	Karnofsky <50 ou Barthel <20		[ ] Sim [ ] Não
**Síndromes geriátricas**	Lesão por pressão	Dados clínicos da anamnese ≥2 recorrentes ou persistentes	[ ] Sim [ ] Não
	Infecções de repetição		
	*Delirium*		
	Disfagia		
	Quedas		
**Sintomas persistentes**	Dor, cansaço, náusea, depressão, ansiedade, sonolência, inapetência, mal-estar, dispneia e insônia	≥2 sintomas (ESAS) recorrentes ou persistentes	[ ] Sim [ ] Não
**Aspectos psicossociais**	Sofrimento emocional ou transtorno adaptativo grave	Detecção do Mal-Estar Emocional >9	[ ] Sim [ ] Não
	Vulnerabilidade social grave	Avaliação social e familiar	[ ] Sim [ ] Não
**Multimorbidade**	≥2 doenças ou condições crônicas avançadas (da lista de indicadores específicos em anexo)		[ ] Sim [ ] Não
**Uso de recursos**	Avaliação da demanda ou intensidade de intervenções	Mais que duas admissões urgentes (não programadas) em 6 meses Aumento da demanda ou intensidade das intervenções (cuidado domiciliar e intervenções de enfermagem)	[ ] Sim [ ] Não
**Indicadores específicos**	Câncer, DPOC, ICC, insuficiência hepática, insuficiência renal, AVC, demência, doenças neurodegenerativas, AIDS e outras doenças avançadas	No Apêndice 2: avaliação dos critérios de gravidade e progressão	[ ] Sim [ ] Não

Traduzido de: Gómez-Batiste X, Martínez-Muñoz M, Blay C, Amblàs J, Vila L, Costa X, et al. Instrumento NECPAL CCOMS-ICO©: identificación de pacientes com enfermedades crónicas evolutivas y necesidades de atenciones y medidas paliativas en servicios de salud y sociales. Centro Colaborador de la OMS para Programas Públicos de Cuidados Paliativos [Internet]. Institut Català d’Oncologia 2011 [cited 2018 May 25]. Available from: http://ico.gencat.cat/web/.content/minisite/ico/professionals/documents/qualy/arxius/doc_necpal_ccoms-ico_instrumento_doc_generalv1_esp_vf_201203.pdf [Tradução autorizada pelo autor]. ABVD: atividades básicas de vida diária; ESAS: Escala de Avaliação de Sintomas de Edmonton; DPOC: doença pulmonar obstrutiva crônica; ICC: insuficiência cardíaca congestiva; AVC: acidente vascular cerebral.

## Apêndice 2

**Table:** Indicadores específicos

Critérios NECPAL de gravidade/progressão/doença avançada*	
**Doença oncológica**	Câncer metastático ou locorregional avançado
	Em progressão em tumores sólidos
	Sintomas persistentes, mal controlados ou refratários, apesar da otimização do tratamento específico
**Doença pulmonar crônica**	Dispneia em repouso ou aos mínimos esforços entre as descompensações
	Restrito ao domicílio com limitação de marcha
	Critérios espirométricos de obstrução grave (VEMS <30%) ou critérios de défice restritivo grave (CVF <40%/DLCO <40%)
	Critérios gasométricos basais de oxigenoterapia domiciliar continuada
	Necessidade de corticoterapia contínua
	Insuficiência cardíaca sintomática associada
**Doença cardíaca crônica**	Dispneia em repouso ou aos mínimos esforços entre as descompensações
	Insuficiência cardíaca NYHA classes III ou IV, doença valvar grave não cirúrgica ou doença coronariana não revascularizável
	Ecocardiograma basal: FE <30% ou HAP grave (PSAP >60)
	Insuficiência renal associada (TFG <60mL/min/1,73m^2^)
	Associação com insuficiência renal e hiponatremia persistente
**Demência**	GDS ≥6c
	Progressão do declínio cognitivo, funcional e/ou nutricional
**Fragilidade**	Índice de Fragilidade do CSHA ≥٠,5
	Avaliação geriátrica integral sugestiva de fragilidade avançada
**Doença neurológica vascular (AVC)**	Durante a fase aguda e subaguda (<3 meses pós-AVC): estado vegetativo persistente ou de mínima consciência >٣ dias
	Durante a fase crônica (>3 meses pós-AVC): complicações médicas repetidas (ou demência com critérios de gravidade pós-AVC)
**Doenças neurológicas degenerativas: ELA, esclerose múltipla e Parkinson**	Piora progressiva da função física e/ou cognitiva
	Sintomas complexos e de difícil controle
	Disfagia persistente
	Transtorno persistente da fala
	Dificuldades crescentes de comunicação
	Pneumonia recorrente por aspiração, dispneia ou insuficiência respiratória
**Doença hepática crônica**	Cirrose avançada estádio Child C (determinado com o paciente fora de complicações ou já as tendo tratado e otimizado o tratamento), MELD-Na >30 ou ascite refratária, síndrome hepatorrenal ou hemorragia digestiva alta por hipertensão portal persistente apesar de otimização do tratamento
	Carcinoma hepatocelular presente, em estádio C ou D
**Doença renal crônica grave**	Insuficiência renal grave (TFG <15mL/minuto) em pacientes que não são candidatos ou que recusam tratamento substitutivo e/ou transplante Finalização da diálise ou falha no transplante

* Usar instrumentos validados de gravidade e/ou prognóstico em função da experiência e evidência; em todos os casos, avaliar também o sofrimento emocional ou o impacto funcional grave em pacientes (e/ou impacto na família) como critério de necessidades paliativas; em todos os casos, avaliar dilemas éticos na tomada de decisões; avaliar sempre a combinação com multimorbidades. VEMS: volume expirante máximo em 1 segundo; CVF: capacidade vital forçada; DLCO: capacidade de difusão do monóxido de carbono; NYHA: *New York Heart Association*; FE: fração de ejeção; HAP: hipertensão arterial pulomonar; PSAP: pressão sistólica da artéria pulmonar; TFG: taxa de filtração glomerular; GDS: Escala de Depressão Geriátrica; CSHA: *Canadian Study of Health and Aging*; AVC: acidente vascular cerebral; ELA: esclerose lateral amiotrófica; MELD-Na: *Model for End-Stage Liver Disease-Sodium.*

**Table:**

Classificação	
Pergunta surpresa	Pergunta surpresa + (não me surpreenderia)
	Pergunta surpresa – (me surpreenderia)
Parâmetros NECPAL	NECPAL + (de 1 a 13 respostas “sim”)
	NECPAL – (nenhum parâmetro assinalado)
**Codificação e registro**	Propor codificação como Paciente com Cronicidade Avançada se pergunta surpresa + e NECPAL +
